# Gene Expression Changes Associated with Nintedanib Treatment in Idiopathic Pulmonary Fibrosis Fibroblasts: A Next-Generation Sequencing and Bioinformatics Study

**DOI:** 10.3390/jcm8030308

**Published:** 2019-03-05

**Authors:** Chau-Chyun Sheu, Wei-An Chang, Ming-Ju Tsai, Ssu-Hui Liao, Inn-Wen Chong, Po-Lin Kuo

**Affiliations:** 1 Division of Pulmonary and Critical Care Medicine, Department of Internal Medicine, Kaohsiung Medical University Hospital, Kaohsiung 807, Taiwan; sheucc@gmail.com (C.-C.S.); 960215kmuh@gmail.com (W.-A.C.); siegfriedtsai@gmail.com (M.-J.T.); chong@kmu.edu.tw (I.-W.C.); 2 Graduate Institute of Clinical Medicine, College of Medicine, Kaohsiung Medical University, Kaohsiung 807, Taiwan; s0970215575@gmail.com; 3 Department of Internal Medicine, School of Medicine, College of Medicine, Kaohsiung Medical University, Kaohsiung 807, Taiwan

**Keywords:** bioinformatics, fibroblasts, *DDX11*, *E2F1*, idiopathic pulmonary fibrosis, microRNA, mRNA, next-generation sequencing, nintedanib, *PLXNA4*

## Abstract

Idiopathic pulmonary fibrosis (IPF) is a chronic, progressive, and fatal interstitial lung disease. Therapeutic options for IPF remain limited. Nintedanib, a tyrosine kinase inhibitor approved for IPF treatment, is known to inhibit fibroblasts proliferation, migration and transformation to myofibroblasts. However, how nintedanib changes gene regulations in IPF has never been systematically investigated. We conducted a next-generation sequencing and bioinformatics study to evaluate the changes of mRNA and miRNA profiles in IPF fibroblasts treated with 2 µM and 4 µM nintedanib, compared to those without treatment. We identified 157 upregulated and 151 downregulated genes and used STRING and DAVID databases for analysis of protein–protein interactions, biological pathways, and molecular functions. We found strong protein–protein interactions within these dysregulated genes, mostly involved in the pathways of cell cycle and mitotic cell cycle. We also discovered 13 potential miRNA–mRNA interactions associated with nintedanib treatment. After validation using miRDB, TargetScan, and RT-qPCR, we identified 4 downregulated genes (*DDX11, E2F1,* *NPTX1,* and *PLXNA4*) which might be repressed by the upregulated hsa-miR-486-3p. According to the proposed functions of *DDX11, E2F1,* and *PLXNA4* reported in previous studies, these gene expression changes together might contribute to decreased proliferation of fibroblasts and decreased angiogenesis in the microenvironment of IPF. Our findings need further studies to confirm.

## 1. Introduction

Idiopathic pulmonary fibrosis (IPF) is a chronic and fatal interstitial lung disease, characterized by rapid progression of lung fibrosis with usual interstitial pneumonia (UIP) as the typical histologic pattern [1,2]. The prognosis of IPF is dismal. The median survival of IPF is around 2–3 years from diagnosis [3,4]. The cause of IPF remains unclear. It is believed that gene-environmental interactions involve in the pathogenesis of IPF. Repetitive environmental stimuli in susceptible individuals lead to aberrant injury-repairing process. Lung epithelial cells are activated and produce pro-fibrotic growth factors and other mediators, which promote epithelial-mesenchymal transition, and activation and differentiation of fibroblasts and myofibroblasts. The activated fibroblasts and myofibroblasts secrete excessive extracellular matrix proteins, resulting in destruction and scaring of lung architecture [5,6,7,8].

To date, therapeutic options for IPF remain limited. Nintedanib and pirfenidone are the only two drugs demonstrated to be effective for IPF [9]. Nintedanib, approved in 2014 in the United States and in 2015 in Europe for IPF treatment, is a multi-target tyrosine kinase inhibitor. The main molecular targets of nintedanib are the fibroblast growth factor receptor (FGFR), the vascular endothelial growth factor receptor (VEGFR), and the platelet-derived growth factor receptor (PDGFR) [10,11]. By competitively and reversibly blocking the receptor tyrosine kinases FGFR, VEGFR, and PDGFR, nintedanib inhibits fibroblasts proliferation, migration and transformation to myofibroblasts [12,13].

IPF is considered as a complex genetic disorder [14]. Several genetic studies has demonstrated the possible dysregulated genes associated with the development of IFP [15,16,17]. Plantier and colleagues used individual data from four microarray studies to compare the transcriptome of IPF versus normal lung fibroblasts. They found downregulated genes in IPF were highly related to inflammation and immunity, and upregulated *CTGF* and *SRF* may play key roles in lung fibrogenesis [18]. How anti-fibrotic agents change gene regulations in IPF, however, has never been systematically investigated. Recently, Kwapiszewska and colleagues compared transcriptomic profiles in IPF lungs obtained from patients treated with or without pirfenidone [19]. They found CEMIP (cell migration-inducing and hyaluronan-binding protein), which has been previously associated with ECM production, was strongly downregulated by pirfenidone treatment.

The rapid evolution of next-generation sequencing (NGS) technology provides a powerful tool for systematic analysis of transcriptomic profiles [20,21]. After creating initial testing strategy and generating sequencing data, investigator could survey for the differential gene expression in the target cells with or without treatment. In order to understand the changes of gene regulation associated with nintedanib treatment in IPF, we conducted a study using the NGS technology and various bioinformatic tools to systematically evaluate the changes of mRNA and miRNA (microRNA) profiles in IPF fibroblasts treated with 2 µM and 4 µM nintedanib, and without treatment. This study not only enhanced current understanding of nintedanib molecular mechanisms, but also provided useful information for future research on pharmacogenomics.

## 2. Experimental Section

### 2.1. Study Design

**Figure 1:**
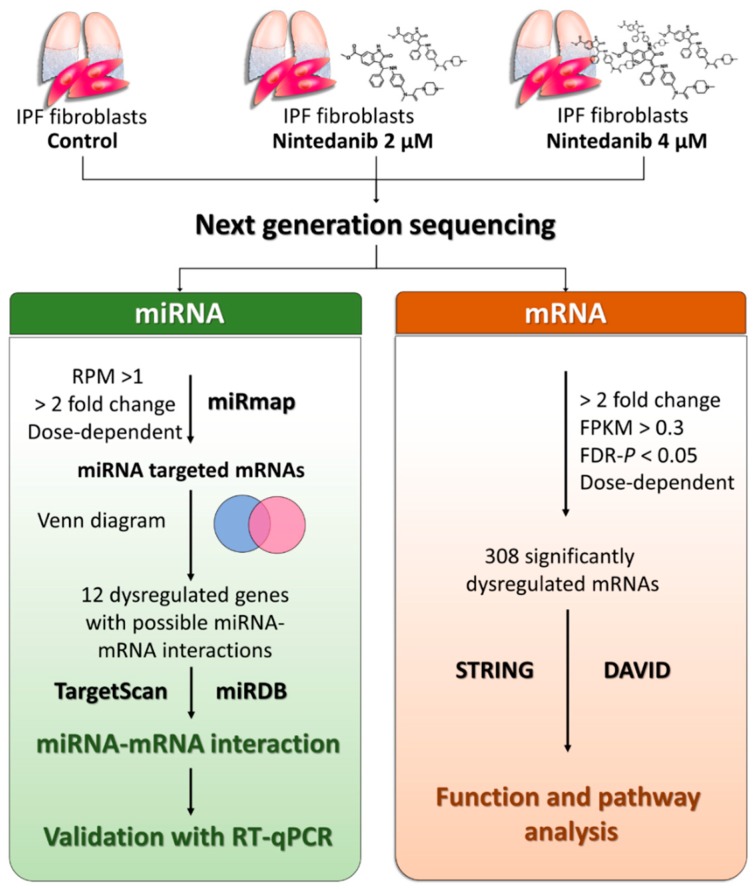
Flowchart of study design. Idiopathic pulmonary fibrosis fibroblasts (the second passage) were cultured with 0.1% dimethyl sulfoxide (DMSO) (control), 2 µM and 4 µM nintedanib for 24 h, and harvested for RNA sequencing and expression profiling using the next-generation sequencing (NGS) platform. Significantly dysregulated mRNAs (>2-fold change, FDR *p* < 0.05, and in a dose-dependent manner) were analyzed with bioinformatic tools including Search Tool for the Retrieval of Interacting Genes (STRING) database and the Database for Annotation, Visualization and Integrated Discovery (DAVID) database to investigate functions and pathways associated with nintedanib treatment. Dysregulated miRNA (microRNA) (>2-fold change and in a dose-dependent manner) were analyzed with miRmap for target prediction, and went through Venn diagram to determine genes with potential miRNA–mRNA interactions. These potential miRNA–mRNA interactions were validated by another prediction database TargetScan and miRDB. Finally, the dysregulations of miRNAs and mRNAs were validated using reverse transcription quantitative polymerase chain reaction (RT-qPCR).

The flowchart of study design is illustrated in Figure 1. IPF fibroblasts (the second passage) were cultured with 0.1% DMSO (control), 2 µM and 4 µM nintedanib for 24 h, and harvested for RNA sequencing and expression profiling using the NGS platform. The significantly upregulated and downregulated mRNAs were analyzed with bioinformatic tools including Search Tool for the Retrieval of Interacting Genes (STRING) database and the Database for Annotation, Visualization and Integrated Discovery (DAVID) database to investigate functions and pathways associated with nintedanib treatment. In addition, the upregulated and downregulated miRNAs were analyzed with miRmap for target prediction, and went through Venn diagram to determine genes with potential miRNA–mRNA interactions. These potential miRNA–mRNA interactions were further validated by another prediction database TargetScan and miRDB. Finally, the dysregulations of miRNAs and mRNAs were validated using reverse transcription quantitative polymerase chain reaction (RT-qPCR).

### 2.2. Cultures of IPF Lung Fibroblasts

Human IPF lung fibroblasts (Disease Human Lung Fibroblasts, Idiopathic Pulmonary Fibrosis. Catalog No. CC-7231), purchased from Lonza Inc. (Walkersville, MD, USA), were incubated at 37 °C in a 5% CO_2_-containing incubator in FGM™-2 Fibroblast Growth Medium-2 Bulletkit™ (Lonza, Catalog No. CC-3132) containing 0.5 mL human fibroblast growth factor-basic (hFGF-B), 0.5 mL insulin, 10 mL fetal bovine serum, and 0.5 mL GA-1000. The medium was changed every 2–3 days and the cells were passaged at 80–90% confluence for the following experiments.

### 2.3. Nintedanib Treatment

Nintedanib (BIBF1120, Catalog No. S1010), purchased from Selleckchem (Houston, TX, USA), was dissolved in dimethyl sulfoxide (DMSO) (Sigma Chemical Co., St. Louis, MO, USA) to obtain various concentrations. The reagents were stored at −20 °C until use in the experiments. In nintedanib-treated cultures, cells were treated with 1 µM, 2 µM, and 4 µM nintedanib for 24 h, 48 h, and 72 h. In control cultures, cells were treated with the carrier solvent (0.1% DMSO). All experiments were performed in triplicate. The selection of nintedanib concentrations of 1–4 µM in this study was mainly based on previous study evaluating the effects of nintedanib on IPF fibroblasts [22].

### 2.4. Cell Morphological Observation

Human IPF lung fibroblasts were incubated in 6 cm plates (1 × 10^5^ cells/plate). When the confluence reached 80%, carrier solvent (0.1% DMSO), 1 µM, 2 µM, or 4 µM nintedanib were added into plates. Cell morphological changes were observed and photographed with an inverted-phase contrast microscope at 24 h, 48 h, and 72 h after nintedanib treatment.

### 2.5. Cell Proliferation Assay

Cell proliferation and viability were assessed using the Premix WST-1 Cell Proliferation Assay System (Takara Bio Inc., Shiga, Japan. Catalog No. MK400), according to the manufacturer’s instructions. In brief, the Premix WST-1 assay measures cell proliferation and cell viability with a colorimetric assay, based on cleavage of tetrazolium salts by mitochondrial dehydrogenase in viable cells. Human IPF lung fibroblasts seeded in the 96-well plates (5 × 10^3^ cells/well) were incubated to confluence of 80% and treated with carrier solvent (0.1% DMSO), 1 µM, 2 µM, or 4 µM nintedanib for 24–72 h. Then 10 µL Premixed WST-1 reagent was added into each well, followed by incubation at 37 °C for 30 to 150 min. Quantification of cell proliferation and viability was measured by detecting the absorbance at 450 nm using a multi-well plate reader (FLX80, BioTek, Winooski, VT, USA). Cell proliferation was expressed as percentage, compared with controls (proliferation % = absorbance in the experimental group/absorbance in the control group × 100%).

### 2.6. Cell Apoptosis Assay

Cell apoptosis was assessed using the Annexin V-FITC Early Apoptosis Detection Kit (Cell signaling Technology, Beverly, MA, USA. Catalog No. 6592S), according to the manufacturer’s instructions. In brief, human IPF lung fibroblasts seeded in the 6 cm plates (1 × 10^5^ cells/well) were incubated to confluence of 80% and treated with carrier solvent (0.1% DMSO), 1 µM, 2 µM, or 4 µM nintedanib for 24–72 h. Harvested cells were double stained with Annexin V and propidium iodide (PI), then analyzed by the BD Accuri™ C6 Plus personal flow cytometer (Becton Dickinson, San Josè, CA, USA). Cell apoptosis was expressed as the percentage of cells with Annexin-V and PI double staining in total cells.

### 2.7. Next-Generation Sequencing (NGS) for miRNA and mRNA Expression Profiling

The expression profiles of miRNAs and mRNAs were examined by using an NGS platform. Total RNA was extracted by TRIzol^®^ Reagent (Thermo Fisher Scientific, Waltham, MA, USA. Catalog No. 15596018) according to the instruction manual. Purified RNA was quantified at OD_260_ nm by using a ND-1000 spectrophotometer (Nanodrop Technologies, Wilmington, DE, USA) and qualitated by using a Bioanalyzer 2100 (Agilent Technologies, Santa Clara, CA, USA) with RNA 6000 LabChip^®^ kit (Agilent Technologies, Santa Clara, CA, USA). The small RNA library construction and deep sequencing were carried out at the biotechnology company Welgene (Taipei, Taiwan). Samples were prepared using Illumina sample preparation kit according to the TruSeq Small RNA Sample Preparation Guide. The 3′ and 5′ adaptors were ligated to total RNA and reverse transcription followed by PCR amplification. The enriched cDNA constructs were size-fractionated and purified on a 6% polyacrylamide gel electrophoresis and the bands containing the 18–40 nucleotide RNA fragments (140–155 nucleotide in length with both adapters). Libraries were sequenced on an Illumina instrument (75 single-end cycles). Sequencing data was processed with the Illumina software. Small RNA sequencing data was also obtained and analyzed. Initially, the sequences generated went through a filtering process to obtain qualified reads. Trimmomatic (version 0.36) [23] was implemented to trim or remove the reads according to the quality score. Qualified reads after filtering low-quality data were analyzed using miRDeep2 [24] to clip the 3′ adapter sequence and discarding reads shorter than 18 nucleotides, before aligning reads to the human genome (version: GRCh38.p10) from UCSC (University of California, Santa Cruz, CA, USA). Only reads that mapped perfectly to the genome five or less times were used for miRNA detection, since miRNAs usually map to a limited number of genomic locations. MiRDeep2 estimates expression levels of known miRNAs, and also identifies novel miRNAs. The miRNAs with low levels (<1 normalized read per million (rpm)) were excluded, and those with >2-fold changes (rpm in 2 µM nintedanib-treated IPF fibroblasts/rpm in controls) in a dose-dependent manner (fold change in 4 µM nintedanib-treated IPF fibroblasts > fold change in 2 µM nintedanib-treated IPF fibroblasts) were considered dysregulated.

For transcriptome sequencing, the Agilent’s SureSelect Strand Specific RNA Library Preparation Kit was used for preparing libraries. Then the libraries were sequenced on a Solexa platform (150 paired-end cycles), using the TruSeq SBS Kit (Illumina, Inc. San Diego, CA, USA). Similar with small RNA sequencing data, reads with low quality score were trimmed and removed using the Trimmomatic (version 0.36). Qualified reads were analyzed using HISAT2 (version 2.1.0) [25], and genes with low expression levels (<0.3 fragment per kilobase of transcript per million mapped reads (FPKM)) in any groups were excluded. *p* values were calculated by cuffdiff (Cufflinks version 2.2.1) with non-grouped samples using the “blind mode”, in which all samples were treated as replicates of a single global "condition" and used to build one model for statistical test [26,27]. To correct for multiple testing, False Discovery Rate (FDR) *p* values were calculated. Genes with FDR *p* values <0.05 (i.e., −log10 (FDR *p*) > 1.3) in 4 µM nintedanib-treated IPF fibroblasts and >2-fold changes (FPKM in 4 µM nintedanib-treated IPF fibroblasts / FPKM in controls) in a dose-dependent manner (fold change in 4 µM nintedanib-treated IPF fibroblasts > fold change in 2 µM nintedanib-treated IPF fibroblasts) were considered as significantly dysregulated genes. For analysis of functions and pathways associated with nintedanib treatment, we uploaded all significantly dysregulated genes into the STRING and DAVID database.

### 2.8. miRmap Database Analysis

miRNA have the ability to repress the expression of protein-coding genes. miRmap is an open-source software library which can provide the prediction of comprehensive miRNA targets (http://mirmap.ezlab.org/) [28]. By calculating the complementary ability of miRNA–mRNA interactions, the putative target genes could be identified. miRmap uses high-throughput experimental data from immunopurification, transcriptomics, proteomics, and polysome fractionation experiments to examine feature correlations and also compare the predictive power. The prediction results provide a list of putative target genes with miRmap score, which is a predictive reference value. In this study, the criteria for selection of putative miRNA targets were miRmap score ≥99.0.

### 2.9. TargetScan Database Analysis

TargetScan predicts the target of miRNA by searching for the presence of conserved 8mer, 7mer, and 6mer sites matching the seed region of each miRNA (http://www.targetscan.org). This is an online database. The results of predictions are ranked by the predicted efficacy of targeting or by their probability of conserved targeting [29]. TargetScan could provide a valuable resource for investigating the role of miRNAs in gene-regulatory networks.

### 2.10. miRDB Database Analysis

miRDB provides web-based microRNA-target prediction and functional annotations (http://mirdb.org) [30,31]. In miRDB, all the targets were predicted by MirTarget. MirTarget was developed by analyzing microRNA-target interactions from high-throughput sequencing experiments. miRDB can predict microRNA targets in five species, including human, mouse, rat, dog, and chicken.

### 2.11. STRING Database Analysis

The functional interactions between expressed proteins in cells are very important and complicated. The Search Tool for the Retrieval of Interacting Genes (STRING) database (https://string-db.org/) has collected and integrated this information, by consolidating known and predicted protein–protein association data for many different organisms. STRING offers the information of the associations include direct (physical) interactions and indirect (functional) interactions. Interactions in STRING are derived from five main sources: Conserved Co-Expressions, High-throughput Lab Experiment, Genomic Context Predictions, Automated Text-mining, and Previous Knowledge in Database [32].

### 2.12. DAVID Database Analysis

The Database for Annotation, Visualization and Integrated Discovery (DAVID) is a powerful tool for gene functional classification (https://david.ncifcrf.gov/) [33]. It integrates Gene Ontology (GO), biological process and Kyoto Encyclopedia of Genes and Genomes (KEGG) pathway. In DAVID database, a list of interesting genes can be classified into clusters of related biological functions, signaling pathways, or diseases by calculating the similarity of global annotation profiles with agglomeration algorithm method. The criteria of Expression Analysis Systematic Explorer (EASE) score in DAVID database is a modified Fisher’s Exact *p* value. The reference score represents how specifically the user genes are involved in the category (for example: signaling pathways). In this study, we selected EASE score = 0.1 as default and define pathways with a Bonferroni *p* value < 0.05 as significant.

### 2.13. RT-qPCR

The expression levels of mRNAs and miRNAs in IPF fibroblasts treated with 0.1% DMSO (control) and 4 µM nintedanib for 24 h were assessed with RT-qPCR. In brief, total RNA was extracted using TRIzol^®^ Reagent (Thermo Fisher Scientific). The mRNAs and miRNAs were reverse transcribed using the PrimeScript RT reagent Kit (Perfect Real Time) (Takara Bio USA, Mountain View, CA, USA) and the Mir-X miRNA First-Strand Synthesis Kit (Takara Bio, USA. Catalog No. 638315), respectively. The mRNA and miRNA expressions were assessed using real-time analysis with Fast SYBR Green Master Mix (Thermo Fisher Scientific. Catalog No. 4385612) catalog number: 4385612)) on the QuantStudio 3 Real-Time PCR System (Thermo Fisher Scientific). The primers used are listed in Supplementary Table S1. The relative expression levels of the mRNAs and miRNAs were normalized to glyceraldehyde-3-phosphate dehydrogenase (GAPDH) and U6 small nuclear RNA (snRNA), respectively. The relative standard curve method (2^−ΔΔCt^) was used to determine relative mRNA and miRNA expression levels.

## 3. Results

### 3.1. Effects of Nintedanib on Cell Morphology, Proliferation, and Apoptosis in IPF Fibroblasts

**Figure 2:**
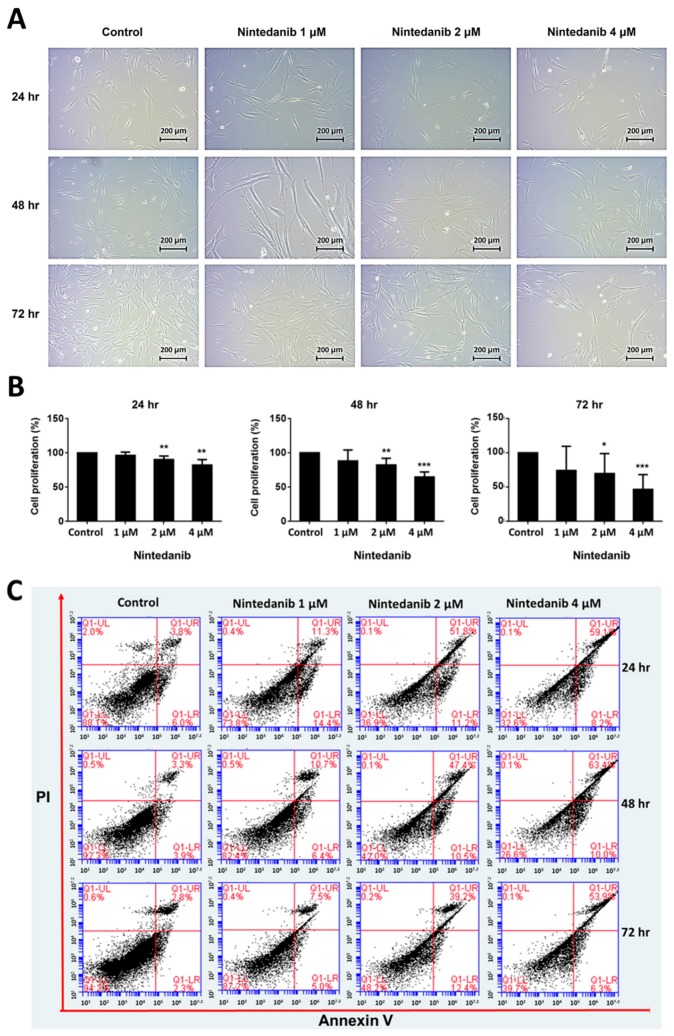
(**A**) Morphological changes of idiopathic pulmonary fibrosis (IPF) fibroblasts after nintedanib treatment. The human IPF lung fibroblasts were treated with 0.1% DMSO (control), 1 µM, 2 µM, and 4 µM nintedanib. The cell morphological changes were observed with an inverted-phase contrast microscope at 24 h, 48 h, and 72 h after nintedanib treatment. (**B**) Inhibition effects of nintedanib on cell proliferation of IPF fibroblasts. The human IPF lung fibroblasts were treated with 0.1% DMSO (control), 1 µM, 2 µM, and 4 µM nintedanib. Cell proliferation was measured with the WST-1 cell proliferation assay at 24 h, 48 h, and 72 h after nintedanib treatment. * *p* < 0.05; ** *p* < 0.01; *** *p* < 0.001. (**C**) Effects of nintedanib on apoptosis of IPF fibroblasts. The human IPF lung fibroblasts were treated with 0.1% DMSO (control), 1 µM, 2 µM, and 4 µM nintedanib. Cell apoptosis was measured with the Annexin V/Propidium Iodide flow cytometry at 24 h, 48 h, and 72 h after nintedanib treatment.

This study aimed to evaluate the gene expression changes associated with nintedanib treatment in IPF fibroblasts. To determine the optimal concentrations and treatment duration of nintedanib, we first performed cell studies to test the effects of 1 µM, 2 µM, or 4 µM nintedanib with treatment duration of 24 h, 48 h, 72 h, on cell morphology, proliferation, and apoptosis in IPF fibroblasts. Figure 2A demonstrates the morphological changes of IPF fibroblasts after nintedanib treatment. As compared to controls, fibroblasts treated with 2 µM and 4 µM nintedanib showed marked inhibition of cell growth and an apparent increase in the formation of vacuoles suggesting autophagy. Figure 2B demonstrates the inhibition effects of nintedanib on cell proliferation of IPF fibroblasts. Inhibition of cell proliferation was observed in IPF fibroblasts treated with 2 µM and 4 µM nintedanib for 24 h, 48 h, and 72 h. Figure 2C demonstrates the effects of nintedanib on apoptosis of IPF fibroblasts. Increased apoptosis and necrosis were observed in IPF fibroblasts treated with 2 µM and 4 µM nintedanib. Based on the results of above experiments, we confirmed the cytostatic and cytotoxic effects of nintedanib on IPF fibroblasts, and decided to evaluate gene expression changes associated with 2 µM and 4 µM of nintedanib treatment. To avoid excessive cell death resulted from over-treatment of nintedanib, the IPF fibroblasts were harvested for deep sequencing after incubation with nintedanib for 24 h.

### 3.2. Overview of Differential Gene Expressions in Nintedanib-Treated IPF Fibroblasts

**Figure 3:**
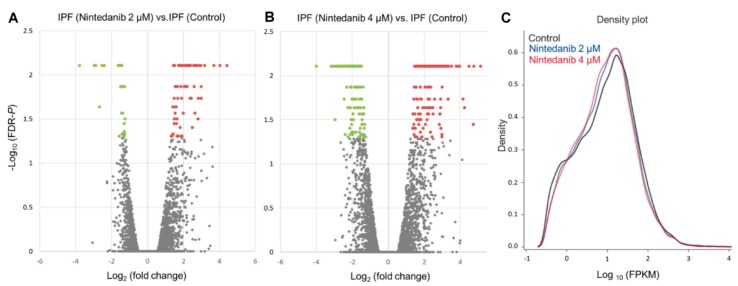
Display of gene expression data in nintedanib-treated IPF fibroblasts. (**A**) Volcano plots of differential gene expression patterns of IPF fibroblasts treated with 2 µM nintedanib versus control. (**B**) Volcano plots of differential gene expression patterns of IPF fibroblasts treated with 4 µM nintedanib versus control. Significantly dysregulated genes, i.e., −log_10_ (FDR *p*) > 1.3 and fold change >2, were shown in red (upregulation) and green (downregulation). (**C**) The density plots illustrate smoothed frequency distribution of the fragments per kilobase of transcript per million mapped reads (FPKM) among controls and IPF fibroblasts treated with 2 µM and 4 µM nintedanib.

The raw sequencing data in this study were uploaded to the GEO repository with the GEO accession number of GSE124786, GSE124787, and GSE124788. The volcano plots of gene expression data in IPF fibroblasts associated with nintedanib treatment were shown in Figure 3A (2 µM nintedanib versus control) and Figure 3B (4 µM nintedanib versus control), with significantly dysregulated genes (fold change >2 and FDR *p* < 0.05) shown in red (upregulation) and green (downregulation). In summary, 84 upregulated genes and 32 downregulated genes were identified in IPF fibroblasts treated with 2 µM nintedanib; while 161 upregulated genes and 206 downregulated genes was identified in IPF fibroblasts treated with 4 µM nintedanib. As expected, more dysregulated genes were identified in IPF fibroblasts treated with 4 µM nintedanib than in fibroblasts treated with 2 µM nintedanib. The density plots of deep sequencing results among 3 groups were showed in Figure 3C. In general, the gene expressions of 4 µM nintedanib-treated IPF fibroblasts were distributed in lower FPKM values and with higher density than 2 µM nintedanib-treated IPF fibroblasts and controls. Among the dysregulated genes identified in 4 µM nintedanib-treated IPF fibroblasts, we excluded dysregulated genes without a dose-dependent manner (defined as fold change in 4 µM nintedanib-treated IPF fibroblasts > fold change in 2 µM nintedanib-treated IPF fibroblasts), leaving a total of 157 upregulated genes and 151 downregulated genes for further analysis (Table S2).

### 3.3. Protein–Protein Interaction, Biological Pathway, and Molecular Function Analysis

**Figure 4:**
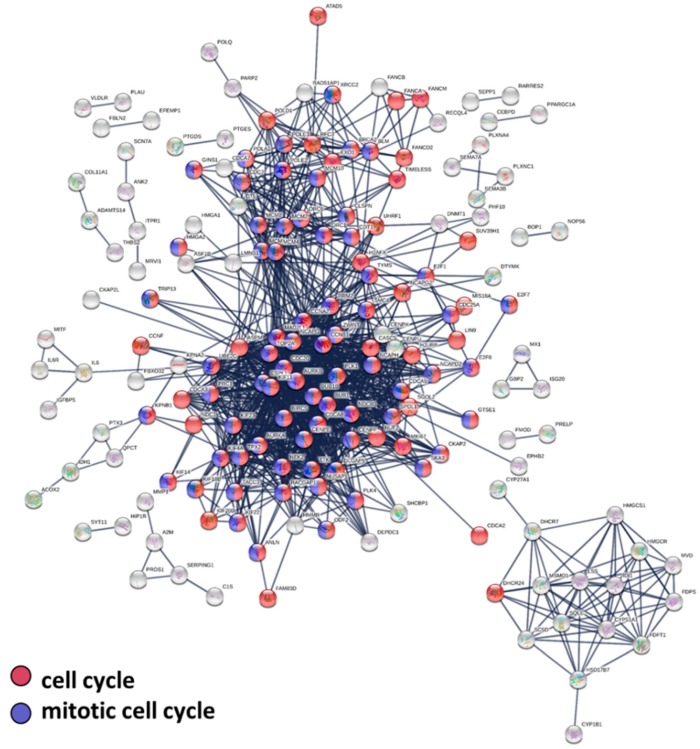
Protein–protein interaction network analysis of dysregulated genes in nintedanib-treated IPF fibroblasts using STRING. The 308 top dysregulated genes (157 upregulated and 151 downregulated) were entered into the Search Tool for the Retrieval of Interacting Genes (STRING) database for protein–protein interaction (PPI) network analysis. The minimum required interaction score was set to the highest confidence (score = 0.900). Nodes without edges are not displayed. This analysis obtained a highly interactive PPI network of 307 nodes and 1227 edges, with PPI enrichment *p* value of <1.0 × 10^−16^. Most genes in the PPI network were related to the following biological pathways: cell cycle (110 genes, shown in red), cell cycle process (94 genes), mitotic cell cycle process (76 genes), and mitotic cell cycle (78 genes, shown in blue). Nodes represent proteins and edges represent protein–protein associations.

The 157 significantly upregulated and 151 significantly downregulated genes were used for protein–protein interactions, biological pathway, and molecular function analysis. Firstly, we uploaded these 308 dysregulated genes into the STRING database for protein–protein interaction (PPI) network analysis. We set the minimum required interaction score as 0.900 (highest confidence) and removed protein nodes without interactions. This analysis obtained a highly interactive PPI network of 307 nodes (nodes represent proteins) and 1227 edges (edges represent protein–protein associations; the expected number of edges was 196), with PPI enrichment *p* value of <1.0 × 10^−16^ (Figure 4). According to gene ontology biological process, most genes in the PPI network were related to the following biological pathways: cell cycle (110 genes, FDR *p* = 1.54 × 10^−46^, shown in red), cell cycle process (94 genes, FDR *p* = 2.12 × 10^−45^), mitotic cell cycle process (76 genes, FDR *p* = 1.82 × 10^−42^), mitotic cell cycle (78 genes, FDR *p* = 1.65 × 10^−41^, shown in blue).

**Figure 5:**
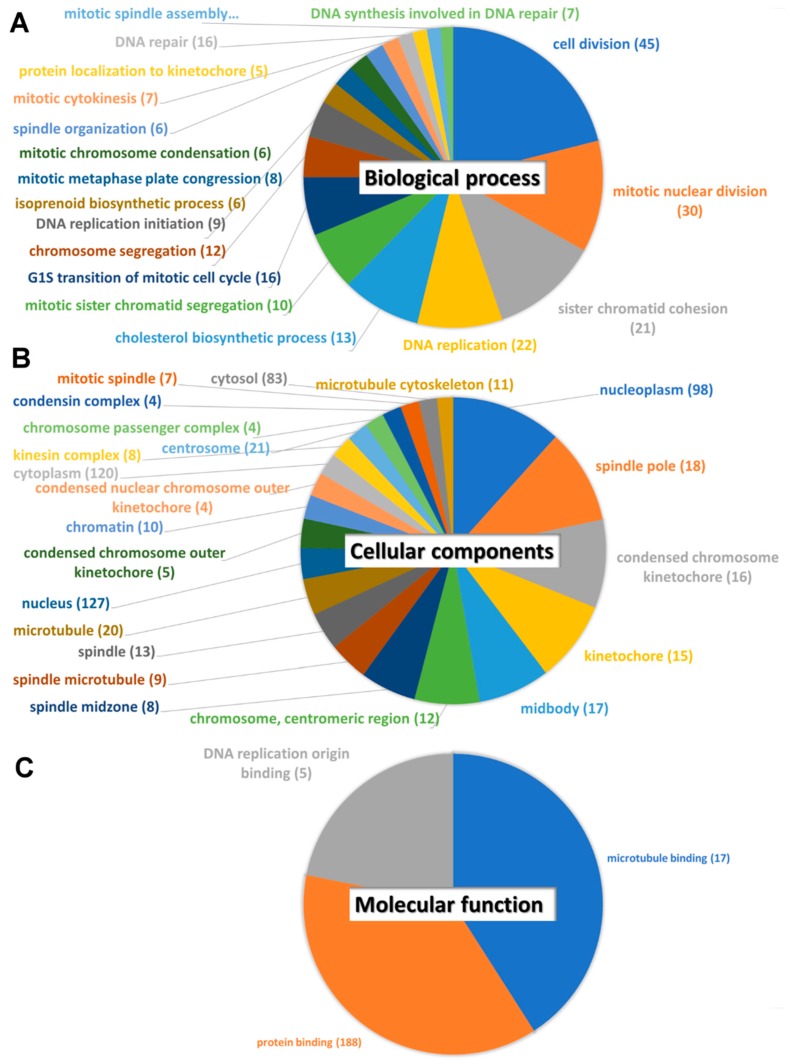
Possible mechanisms of the dysregulated genes in nintedanib-treated IPF fibroblasts analyzed by DAVID. (**A**) Biological process. (**B**) Cellular components. (**C**) Molecular functions. The counts of involved genes were showed as the number in. Pathways with a Bonferroni *p* value < 0.05 were considered significant. The area of each pathway reflected the significant level, based on their −log_10_ (Bonferroni *p*) values.

Secondly, we used DAVID to analyze possible mechanisms of the top dysregulated genes in IPF fibroblasts related to nintedanib treatment. In biological process (Figure 5A), cell division (45 genes were involved), mitotic nuclear division (30 genes), sister chromatid cohesion (21 genes), and DNA replication (22 genes) had the most significant status. In cellular components (Figure 5B), nucleoplasm (98 genes), spindle pole (18 genes), and condensed chromosome kinetochore (16 genes) had the most significant status. In molecular functions (Figure 5C), microtubule binding (17 genes) and protein binding (188 genes) had the most significant status.

### 3.4. Dysregulated Genes with Potential miRNA–mRNA Interactions in Nintedanib-Treated IPF Fibroblasts

**Figure 6:**
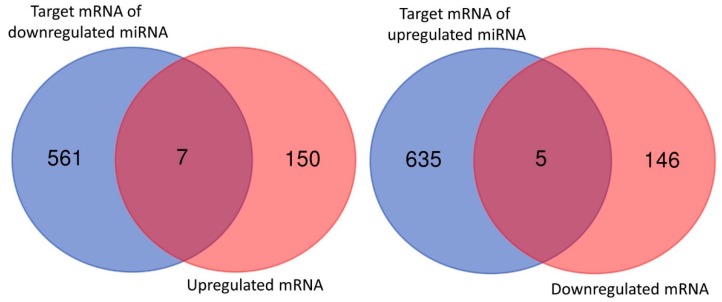
Identification of dysregulated genes with potential miRNA–mRNA interactions in nintedanib-treated IPF fibroblasts. The Venn diagram analysis of predicted targets of dysregulated miRNAs and dysregulated mRNAs identified 7 upregulated genes and 5 downregulated genes with potential miRNA–mRNA interactions.

Using the miRmap database with a criteria of miRmap score ≥99.0, we predicted 568 target mRNAs from 46 significantly downregulated miRNAs and 640 target mRNAs from 36 significantly upregulated miRNAs. Venn diagram analysis of the 568 predicted targets (of 46 downregulated miRNAs) and 157 upregulated mRNAs identified 7 upregulated genes with potential miRNA–mRNA interactions in nintedanib-treated IPF fibroblasts; while analysis of the 640 predicted targets (of 36 upregulated miRNAs) and 151 downregulated mRNAs identified 5 downregulated genes with potential miRNA–mRNA interactions (Figure 6). Table 1 shows the 13 potential miRNA–mRNA interactions, involving 5 miRNAs and 12 genes, associated with nintedanib treatment. Only interactions of hsa-miR-486-3p with *DDX11*, *E2F1, NPTX1,* and *PLXNA4*, and interaction of hsa-miR-92a-1-5p with *SLC25A23* were validated in both TargetScan and miRDB database. The details of possible binding sites in these 5 validated miRNA–mRNA interactions were provided in the Table S3.

**Figure 7:**
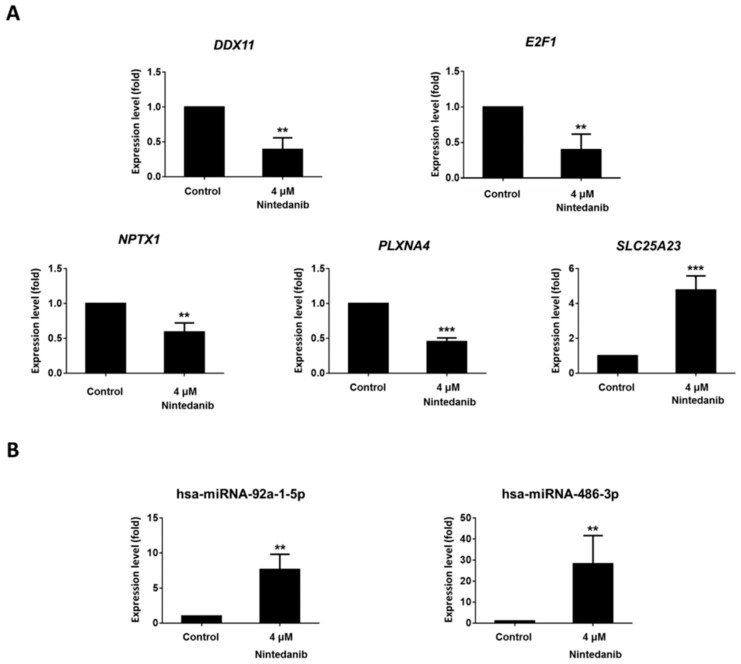
The expression levels of (**A**) mRNAs and (**B**) miRNAs in IPF fibroblasts treated with 0.1% DMSO (control) and 4 µM nintedanib assessed with RT-qPCR. The expression levels in nintedanib-treated IPF fibroblasts were compared to the expression levels in controls, expressed as mean ± standard error of the mean, and analyzed with Student’s *t*-test. ** *p* < 0.01; *** *p* < 0.001.

To further validate the results from NGS analysis, the expression levels the mRNA and miRNA involved in the 5 potential miRNA–mRNA interactions were assessed with RT-qPCR in another sets of IPF fibroblasts treated with 0.1% DMSO (control) and 4 µM nintedanib for 24 h. For mRNAs, the expression levels of *DDX11*, *E2F1, NPTX1,* and *PLXNA4* were significantly lower in nintedanib-treated IPF fibroblasts than in controls, and the expression levels of *SLC25A23* were significantly higher in nintedanib-treated IPF fibroblasts than in controls (Figure 7A). For miRNAs, the expression levels of both hsa-miR-486-3p and hsa-miR-92a-1-5p were significantly higher in nintedanib-treated IPF fibroblasts than in controls (Figure 7B). The dysregulations of these mRNA and miRNA identified from NGS analysis were all validated in RT-qPCR, except for hsa-miR-92a-1-5p.

## 4. Discussion

IPF is the most common among idiopathic interstitial pneumonias. The incidence of IPF ranges between 0.22 and 17.4 per 100,000 population and the patients with IPF are usually in the sixth or seventh decade of life [12]. Because of the increasing incidence and the lethal nature of this disease, more attention has been drawn to develop effective treatment for IPF. Nintedanib, a multi-target tyrosine kinase inhibitor, has been demonstrated to retardate the disease progression in IPF patients [34]. Nintedanib is able to inhibit TGF-β signaling, downregulate extracellular matrix (ECM) gene/protein expression and promote noncanonical autophagy [22]. To further investigate the molecular mechanisms associated with nintedanib treatment in IPF, we used NGS to scan mRNA and miRNA changes in primary human IPF fibroblasts treated with 2 µM and 4 µM nintedanib, compared to those without treatment. We identified 151 significantly upregulated genes and 157 significantly downregulated genes associated with nintedanib treatment in a dose-dependent manner. These 308 dysregulated genes were then analyzed in different bioinformatic tools for their protein–protein interactions, biological pathways, and molecular functions.

The STRING analysis showed strong protein–protein interactions within the top dysregulated genes. Interestingly, most genes in the PPI network were related to biological pathways of cell cycle, cell cycle process, mitotic cell cycle process, and mitotic cell cycle. The DAVID analysis revealed that the role of nintedanib in treating IPF fibroblast might most possibly relate to the biological process of cell division, mitotic nuclear division, sister chromatid cohesion, and DNA replication. We also identified 7 upregulated genes and 5 downregulated genes with potential miRNA–mRNA interactions. After validation in TargetScan and miRDB, upregulated *SLC25A23* (might be de-repressed by downregulated hsa-miR-92a-1-5p) and downregulated *DDX11, E2F1, NPTX1,* and *PLXNA4* (might be repressed by the upregulated hsa-miR-486-3p) were validated as the dysregulated genes with potential miRNA–mRNA interactions associated with nintedanib treatment. The dysregulations of these mRNAs and miRNAs were further validated with RT-qPCR, except for hsa-miR-92a-1-5p.

Our data showed that *DDX11* was downregulated after nintedanib treatment. *DDX11* is the predicted target of hsa-miR-486-3p, which is upregulated in nintedanib-treated IPF fibroblasts. *DDX11*, also known as CHL-1, is able to promote cell proliferation, metastasis, and migration in human glioma cells [35]. Although no previous study investigating the role of *DDX11* in IPF fibroblasts, our data suggest that nintedanib may suppress *DDX11* in IPF fibroblasts and therefore suppress the proliferation of IPF fibroblasts. According to our NGS data, the expression change of *DDX1* in nintedanib-treated IPF fibroblasts was in a dose-dependent manner. Therefore, we hypothesized that nintedanib might decreased proliferation of IPF fibroblasts through regulation of hsa-miR-486-3p-*DDX11*.

We found that nintedanib treatment was associated with downregulation of *PLXNA4*. *PLXNA4* is also the predicted target of the upregulated hsa-miR-486-3p in nintedanib-treated IPF fibroblasts. PLXNA4 may promote progression and angiogenesis of tumor, possibly by enhancement of VEGF and bFGF signaling pathways [36]. Downregulation of *PLXNA4* may be associated with decreased angiogenesis. Therefore, our data suggest the changed expressions of hsa-miR-486-3p-*PLXNA4* associated with nintedanib treatment may contribute to decreased angiogenesis in IPF.

Our data also showed that *E2F1* was downregulated after nintedanib treatment. Similar to *DDX11* and *PLXNA4*, *E2F1* is the predicted target of hsa-miR-486-3p. E2F1 protein is a member of the E2F family of transcription factors. The E2F1 transcription factor play critical roles in the regulation of cell cycle and apoptosis [37]. It is considered as one of the most important protein required for entering into the S phase during cell cycle progression [38]. Several growth factors including epidermal growth factor (EGF), keratinocyte growth factor (KGF), and VEGF exert their proliferating biological activity by increasing expression of *E2F1* [37,39,40,41]. It is not surprising to know that treatment with nintedanib, which targets many growth factor receptors, was associated with downregulation of *E2F1*. Our interaction analysis suggests that upregulation of hsa-miR-486-3p might have an interaction with downregulation of *E2F1* in nintedanib-treated IPF fibroblasts. However, this hsa-miR-486-3p-*E2F1* interaction was predicted from the bioinformatic database. How hsa-miR-486-3p, E2F1 transcription factors, and growth factors interact with each other in nintedanib treatment need further studies to confirm and elucidate.

Another downregulated gene *NPTX1* was also the predicted target of hsa-miR-486-3p. The protein encoded by *NPTX1* is a member of the neuronal pentraxin family. The expression of this gene is mainly localized to the nervous system [42]. The only upregulated gene with miRNA–mRNA interaction found in this study was *SLC25A23*, which might have an interaction with the downregulated miRNA hsa-miR-92a-1-5p based on our bioinformatic prediction. *SLC25A23* is a member of the mitochondrial transporter family SLC25 embedded in the inner mitochondrial membrane [43]. *SLC25A23* has been shown to augment mitochondrial matrix Ca^2+^ influx, and may induce oxidative stress-mediated cell death [44]. Although the function of this gene remains largely unknown, it is possible that nintedanib treatment downregulated hsa-miR-92a-1-5p which in turn de-repressed *SLC25A23* and enhance cell death of fibroblasts. However, the downregulation of hsa-miR-92a-1-5p in nintedanib-treated IPF fibroblasts was not confirmed by the RT-qPCR method.

**Figure 8:**
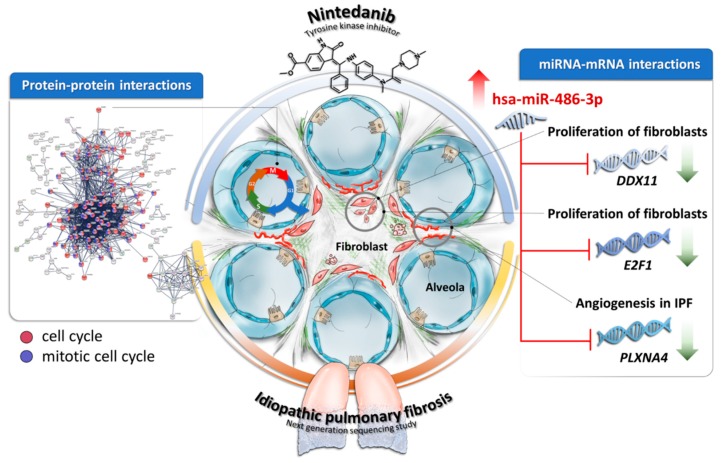
The schematic summary of gene expression changes and possible molecular mechanisms associated with nintedanib treatment in IPF fibroblasts. Strong protein–protein interactions was found within the dysregulated genes in nintedanib-treated IPF fibroblasts, with most genes involved in pathways of cell cycle and mitotic cell cycle. The upregulation of hsa-miR-486-3p might be associated with decreased expression of *DDX11, E2F1,* and *PLXNA4*. These gene expression changes together might contribute to decreased proliferation of fibroblasts and decreased angiogenesis in the microenvironment of IPF.

To the best of our knowledge, this is the first study using NGS and bioinformatics to scan mRNA and miRNA changes following nintedanib treatment. We identified 308 significantly dysregulated genes associated with nintedanib treatment in IPF fibroblasts. We found strong protein–protein interactions within these dysregulated genes, mostly involved in the pathways of cell cycle and mitotic cell cycle. We also identified 1 upregulated and 4 downregulated genes with potential miRNA–mRNA interactions. According to our data from nintedanib-treated IPF fibroblasts and the literatures, we proposed that upregulation of hsa-miR-486-3p following nintedanib treatment might be associated with decreased expression of *DDX11, E2F1,* and *PLXNA4*. These gene expression changes together might contribute to decreased proliferation of fibroblasts and decreased angiogenesis in the microenvironment of IPF (Figure 8). Our findings improve current understandings of molecular mechanisms of nintedanib treatment in IPF. Our approach and results may also provide useful information for future research on nintedanib pharmacogenomics. Nevertheless, there remain several limitations in this study. Firstly, as nintedanib is known to interfere with VEGFR, FGFR, and PDGFR, and potentially also with TGF-β signaling, its therapeutic effects will likely be observed when cells are stimulated with VEGF, FGF, PDGF, and/or TGF-β. The culture medium used in this study contains only FGF, but VEGF and PDGF might also be present in the fetal bovine serum in unknown quantities. The FGF-containing culture condition might somewhat explain our findings that nintedanib-induced gene expression changes were mostly related to pathways of cell cycle, instead of pathways of TGF-β signaling, ECM, and autophagy. Considering that results would be different when fibroblasts were stimulated with different growth factors in the medium, plus there is no perfect culture condition mimicking the true in vivo microenvironment of IPF lung, we decided to cultivate the IPF lung fibroblasts using the standard condition as suggested by Lonza. Secondly, the dysregulated miRNAs, mRNAs and their interactions found in this study were based on primary IPF lung fibroblasts, RNA deep sequencing, bioinformatics, and RT-qPCR. The dysregulations of these genes need to be confirmed by other methods and in more clinical samples. The relationships between miRNAs and gene expressions found in this study also need be confirmed in future cell-based assays.

## 5. Conclusions

We identified 308 significantly dysregulated genes associated with nintedanib treatment in IPF fibroblasts. We found strong protein–protein interactions within these dysregulated genes, with most genes involved in the pathways of cell cycle and mitotic cell cycle. In IPF fibroblasts, we found nintedanib treatment was associated with upregulation of hsa-miR-486-3p, which might repress *DDX11, E2F1,* and *PLXNA4* expressions. These together might contribute to decreased proliferation of fibroblasts and decreased angiogenesis in the microenvironment of IPF. However, this is a screening study using primary IPF lung fibroblasts and bioinformatics approaches. Our findings need further studies to confirm.

## Figures and Tables

**Table 1:** Potential miRNA–mRNA interactions associated with nintedanib treatment validated with miRmap, TargetScan and miRDB.

miRNA	Fold Change	Gene Symbol	Fold Change	miRmap Score	TargetScan Total Context++ Score	miRDB
hsa-miR-486-3p	2.31	*DDX11*	−4.05	99.389	−0.50	59
hsa-miR-486-3p	2.31	*E2F1*	−5.78	99.597	−0.85	65
hsa-miR-486-3p	2.31	*NPTX1*	−2.89	99.908	−0.62	95
hsa-miR-486-3p	2.31	*PLXNA4*	−6.94	99.700	−0.18	70
hsa-miR-92a-1-5p	−2.71	*SLC25A23*	2.68	99.527	−1.27	65
hsa-miR-1275	−4.74	*PRELP*	3.87	99.724		65
hsa-miR-1275	−4.74	*HRK*	5.36	99.881		87
hsa-miR-100-3p	−2.52	*GJA3*	3.04	99.326	−0.39	
hsa-miR-1275	−4.74	*ZBED3*	3.46	99.669	−0.94	
hsa-miR-1275	−4.74	*ANTXR1*	2.69	99.535	−0.66	
hsa-miR-486-3p	2.31	*SUV39H1*	−4.35	99.421	−0.47	
hsa-miR-1275	−4.74	*MBP*	8.97	99.886		
hsa-miR-326	4.64	*PLXNA4*	−6.94	99.932		

Potential miRNA–mRNA interactions validated in both TargetScan and miRDB were shown in bold. Fold change indicates the expression change in 4 µM nintedanib-treated IPF fibroblasts, compared to controls. The context++ score for a specific site is the sum of the contribution of 14 features. The total context++ score is calculated by summing the context++ scores for the sites to the representative miRNA.

## Supplementary Materials

The following are available online at https://www.mdpi.com/2077-0383/8/3/308/s1, Table S1: The primers used in RT-qPCR, Table S2: Dysregulated genes in nintedanib-treated IPF fibroblast, Table S3: Possible binding sites of the 5 potential miRNA–mRNA interactions.
