# Supplementary material for: Gene Expression Changes Associated with Nintedanib Treatment in Idiopathic Pulmonary Fibrosis Fibroblasts: A Next-Generation Sequencing and Bioinformatics Study
Source: J Clin Med. 2019 Mar 5;8(3):308. doi: 10.3390/jcm8030308 (PMC6462954; doi:10.3390/jcm8030308)
Supplement: Supplementary file 1 [file jcm-08-00308-s001.pdf]

Table S1. The primers used in RT-qPCR.

**Gene**	**5′ to 3′**
*DDX11*_Forward	AGGACGACTGAAGCTCTTGC
*DDX11*_Reverse	CACAGCTGGATTCAGGAGCA
*E2F1*_Forward	CGCCATCCAGGAAAAGGTGT
*E2F1*_Reverse	ATGTCATAGATGCGCCGCTT
*NPTX1*_Forward	CCAACGAGCTGGTCCTCATT
*NPTX1*_Reverse	GTGATAGGGCGCCAAGTTCT
*PLXNA4*_Forward	TCAAAGGAGACCGGCATGAC
*PLXNA4*_Reverse	CTGGAAGGAGCAGGTCTTGG
*SLC25A23*_Forward	GATGTGACGTAGCAGACCCA
*SLC25A23*_Reverse	TGGTGGCTTCAGCAGTACAG
*GAPDH*_Forward	GAGTCAACGGATTTGGTCGT
*GAPDH*_Reverse	TTGATTTTGGAGGGATCTCG
**miRNA**	**5′ to 3′**
has-miR-92a-1-5p	AGGTTGGGATCGGTTGCAATGCT
has-miR-486-3p	CGGGGCAGCTCAGTACAGGAT

Table S2. Dysregulated genes in nintedanib-treated IPF fibroblast.

Upregulated Genes									
	FPKM			Nintedanib 2 μM vs. Control			Nintedanib 4 μM vs. Control		
Gene Symbol	Control	Nintedanib 2 μM	Nintedanib 4 μM	Ratio	*P* value	FDR *P*	Ratio	*P* value	FDR *P*
CYP51A1	85.111	246.905	350.467	2.90098	0.0002	0.02302	4.11776	0.00005	0.00781
IL32	0.6016	2.36249	3.28585	3.92702	0.00025	0.02717	5.46187	0.00005	0.00781
PLEKHB1	0.03776	1.03393	1.53624	27.3832	0.0001	0.01351	40.6867	0.00005	0.00781
RAB27B	0.81566	4.15225	4.80502	5.09066	0.00015	0.01843	5.89095	0.00005	0.00781
MSMO1	83.2235	314.354	428.344	3.77723	0.00005	0.00781	5.14691	0.00005	0.00781
COL11A1	1.5869	12.1888	11.3932	7.68089	0.00005	0.00781	7.17953	0.00005	0.00781
GUCY1B3	2.47552	6.82968	12.6249	2.75889	0.00805	0.25699	5.0999	0.00005	0.00781
IDI1	59.8703	182.112	294.135	3.04178	0.0052	0.19776	4.91287	0.00005	0.00781
LMCD1	11.8082	46.4574	57.2643	3.93433	0.00025	0.02717	4.84954	0.00005	0.00781
FRY	3.34931	11.879	12.8797	3.5467	0.00005	0.00781	3.84548	0.00005	0.00781
MAP2	5.64117	28.0845	35.1711	4.97849	0.00005	0.00781	6.23472	0.00005	0.00781
FDFT1	82.1629	185.723	227.837	2.26042	0.0015	0.0899	2.77299	0.00005	0.00781
SLC7A8	4.82707	15.5507	31.1599	3.22156	0.00055	0.04936	6.45524	0.00005	0.00781
PALMD	0.89599	4.74914	6.65261	5.30043	0.00015	0.01843	7.42485	0.00005	0.00781
KCNK6	4.49098	15.6689	16.5609	3.48897	0.00005	0.00781	3.68759	0.00005	0.00781
PLA2G3	0.32468	2.54314	3.71021	7.83266	0.0001	0.01351	11.4271	0.00005	0.00781
HMOX1	22.6002	52.6263	80.8803	2.32858	0.00085	0.06557	3.57874	0.00005	0.00781
ABHD4	8.54186	18.2277	27.0067	2.13393	0.00495	0.19368	3.16169	0.00005	0.00781
CRISPLD2	5.27383	13.9721	20.9472	2.64933	0.0005	0.04658	3.97191	0.00005	0.00781
QPRT	8.6699	24.9865	28.9918	2.88198	0.00025	0.02717	3.34396	0.00005	0.00781
RASL12	0.3035	1.40887	2.54339	4.64203	0.00115	0.0759	8.38011	0.00005	0.00781
SQLE	50.292	125.024	180.09	2.48596	0.00055	0.04936	3.58089	0.00005	0.00781
ITGB8	3.49863	12.8903	15.3215	3.68439	0.00005	0.00781	4.37929	0.00005	0.00781
PRUNE2	2.64214	11.298	21.8766	4.27608	0.0001	0.01351	8.27988	0.00005	0.00781
SC5D	43.9941	103.822	132.397	2.35991	0.00165	0.09584	3.00943	0.00005	0.00781
HMGCS1	60.0461	283.065	519.204	4.71413	0.00005	0.00781	8.64676	0.00005	0.00781
NME5	0.0001	1.41923	2.59631	14192.3	0.00005	0.00781	25963.1	0.00005	0.00781
HMGCR	37.1334	92.0897	159.768	2.47997	0.0007	0.05725	4.30254	0.00005	0.00781
EFEMP1	21.7203	57.2904	77.8266	2.63764	0.0002	0.02302	3.58313	0.00005	0.00781
QPCT	1.70109	9.0651	10.8714	5.32899	0.00015	0.01843	6.39084	0.00005	0.00781
DHCR24	97.4412	243.228	276.696	2.49615	0.00065	0.05472	2.83962	0.00005	0.00781
PLPPR4	4.14454	20.8544	28.4783	5.03178	0.00005	0.00781	6.87128	0.00005	0.00781
TCF21	7.49341	46.17	66.9578	6.16141	0.00005	0.00781	8.93556	0.00005	0.00781
C1orf198	13.392	37.8735	40.9468	2.82807	0.00005	0.00781	3.05756	0.00005	0.00781
NR4A3	1.44922	8.44807	22.3449	5.82939	0.00005	0.00781	15.4186	0.00005	0.00781
AVPI1	4.98145	22.3648	28.2743	4.48962	0.00005	0.00781	5.67592	0.00005	0.00781
FABP3	19.1573	171.406	268.067	8.94729	0.00005	0.00781	13.9929	0.00005	0.00781
NR4A1	6.00173	22.6834	33.4358	3.77948	0.00005	0.00781	5.57103	0.00005	0.00781
KCNJ2	3.61596	12.941	15.6956	3.57886	0.0001	0.01351	4.34065	0.00005	0.00781
TMEM255A	0.8491	4.42398	5.65901	5.21019	0.00005	0.00781	6.66471	0.00005	0.00781
MASP1	2.9766	11.0434	18.8306	3.71007	0.00015	0.01843	6.32621	0.00005	0.00781
ACSS2	10.751	31.6077	56.2667	2.93998	0.0002	0.02302	5.23362	0.00005	0.00781
RAMP1	2.46885	11.2088	18.3691	4.54009	0.0001	0.01351	7.44035	0.00005	0.00781
SYT11	22.6546	74.6388	82.3654	3.29464	0.00005	0.00781	3.6357	0.00005	0.00781
EPSTI1	0.54844	3.34983	3.84466	6.10798	0.00005	0.00781	7.01024	0.00005	0.00781
KRT7	2.68951	12.2456	10.7174	4.5531	0.00005	0.00781	3.98489	0.00005	0.00781
IL6	0.5836	9.50339	15.8866	16.2841	0.00005	0.00781	27.2217	0.00005	0.00781
SCN7A	0.43332	9.22359	15.2088	21.2858	0.00005	0.00781	35.0981	0.00005	0.00781
CYP1B1	28.9011	132.36	131.272	4.57976	0.00005	0.00781	4.54211	0.00005	0.00781
SLC40A1	2.81819	20.8316	38.5491	7.39184	0.00005	0.00781	13.6787	0.00005	0.00781

GABARAPL1	10.6071	46.6337	68.1393	4.39646	0.00005	0.00781	6.42393	0.00005	0.00781
SNCA	1.06913	6.55458	10.146	6.13076	0.00025	0.02717	9.48996	0.00005	0.00781
TTBK1	0.02723	0.56922	0.67189	20.9043	0.00015	0.01843	24.6746	0.00005	0.00781
PTGES	0.80834	10.3906	18.0879	12.8543	0.00005	0.00781	22.3766	0.00005	0.00781
SERPING1	11.7528	31.1492	40.9299	2.65036	0.0024	0.12395	3.48257	0.00005	0.00781
ITPR1	2.14873	6.57691	8.99693	3.06084	0.00125	0.0795	4.18709	0.00005	0.00781
FBXO32	2.50888	5.19172	16.2372	2.06934	0.0267	0.48222	6.47189	0.00005	0.00781
TSC22D3	3.53944	16.0225	26.1375	4.52685	0.0001	0.01351	7.38464	0.00005	0.00781
COX6B2	0.0001	0.42945	0.66676	4294.52	0.00005	0.00781	6667.57	0.00005	0.00781
IL6R	2.07724	6.09374	8.01432	2.93358	0.00085	0.06557	3.85816	0.00005	0.00781
FDPS	100.824	236.872	306.508	2.34936	0.0051	0.19594	3.04003	0.00005	0.00781
FGFR4	0.41418	3.24768	4.19507	7.84121	0.00015	0.01843	10.1286	0.00005	0.00781
DMKN	1.63121	10.8443	25.5677	6.64801	0.00005	0.00781	15.6741	0.00005	0.00781
ITIH3	0.0001	0.62354	0.37469	6235.43	0.00005	0.00781	3746.92	0.00005	0.00781
GBP2	4.14821	13.3421	16.2216	3.21635	0.0007	0.05725	3.91051	0.00005	0.00781
PBXIP1	8.82626	22.5696	30.8042	2.5571	0.00425	0.17556	3.49006	0.00005	0.00781
LMOD1	6.41129	20.1124	23.6959	3.13703	0.0001	0.01351	3.69596	0.00005	0.00781
FBLN2	2.55628	6.40659	8.29674	2.50622	0.00155	0.09222	3.24563	0.00005	0.00781
FAM198B	2.59285	15.5109	19.7946	5.98218	0.00005	0.00781	7.6343	0.00005	0.00781
ELOVL7	0.0001	0.33987	0.46469	3398.65	0.00005	0.00781	4646.94	0.00005	0.00781
ALDH1A1	10.0967	43.6441	63.0852	4.32261	0.00005	0.00781	6.2481	0.00005	0.00781
TMEM130	1.00813	6.41711	9.7974	6.36536	0.00005	0.00781	9.71839	0.00005	0.00781
NNMT	22.0815	68.2184	83.9306	3.08939	0.00055	0.04936	3.80095	0.00005	0.00781
MVD	26.2264	75.5978	118.777	2.88251	0.0001	0.01351	4.52891	0.00005	0.00781
PCSK9	0.22696	0.847	1.8135	3.73193	0.00525	0.19874	7.99036	0.00005	0.00781
MN1	0.43611	2.14195	4.07207	4.91152	0.00005	0.00781	9.33732	0.00005	0.00781
SYNPO	2.55124	10.509	11.7668	4.11917	0.00005	0.00781	4.61219	0.00005	0.00781
ISG20	0.46154	1.85702	3.30114	4.0235	0.0096	0.28438	7.15238	0.00005	0.00781
SYNPO2	7.52423	27.3958	27.2474	3.64101	0.00005	0.00781	3.62129	0.00005	0.00781
DHCR7	32.4392	96.4099	139.98	2.97202	0.0001	0.01351	4.31515	0.00005	0.00781
PODN	4.71906	16.2485	16.0535	3.44316	0.00005	0.00781	3.40184	0.00005	0.00781
A2M	1.68293	6.965	13.6387	4.13862	0.0007	0.05725	8.10414	0.00005	0.00781
MAF	5.19774	19.6118	34.114	3.77314	0.00005	0.00781	6.56324	0.00005	0.00781
OLFML1	17.0525	44.8176	51.7319	2.62821	0.00065	0.05472	3.03368	0.00005	0.00781
CEND1	1.40638	9.08861	10.7633	6.46241	0.00005	0.00781	7.65319	0.00005	0.00781
THBS2	11.9091	34.4398	43.7726	2.89189	0.0001	0.01351	3.67556	0.00005	0.00781
TPRG1	0.0001	0.65649	0.44839	6564.87	0.00005	0.00781	4483.86	0.00005	0.00781
PRELP	5.94306	20.3331	22.9857	3.42132	0.00005	0.00781	3.86765	0.00005	0.00781
LITAF	56.8891	152.129	210.397	2.67413	0.00005	0.00781	3.69837	0.00005	0.00781
NLGN3	0.18641	1.28997	1.90115	6.92003	0.0002	0.02302	10.1987	0.00005	0.00781
DPP4	18.3098	51.287	68.9147	2.80107	0.0003	0.03149	3.76382	0.00005	0.00781
FAM49A	1.18426	4.43354	5.75523	3.74372	0.00055	0.04936	4.85977	0.00005	0.00781
MBP	0.91393	4.53777	8.20287	4.96512	0.00005	0.00781	8.97538	0.00005	0.00781
LBH	59.1584	222.128	211.065	3.7548	0.00005	0.00781	3.56779	0.00005	0.00781
CEBPD	3.52652	12.8432	18.3278	3.64189	0.00005	0.00781	5.19713	0.00005	0.00781
SLC12A8	1.05204	6.2713	8.26196	5.96109	0.0001	0.01351	7.85328	0.00005	0.00781
HBD	0.0001	0.56368	0.97227	5636.83	0.00015	0.01843	9722.66	0.00005	0.00781
C1QTNF5	4.678	19.1955	22.4531	4.10336	0.0001	0.01351	4.79972	0.00005	0.00781
MIAT	0.8635	4.48866	6.86352	5.1982	0.0003	0.03149	7.94846	0.00005	0.00781
SELENOP	16.1881	106.181	163.901	6.5592	0.00005	0.00781	10.1248	0.00005	0.00781
NATD1	1.09513	2.79433	5.3477	2.5516	0.00645	0.22565	4.88316	0.00005	0.00781
TRO	2.78669	9.69767	11.6692	3.48	0.0004	0.03952	4.18748	0.0001	0.01351
NTN4	4.67257	19.2037	19.2069	4.10988	0.0001	0.01351	4.11056	0.0001	0.01351
IGFBP5	241.188	787.991	834.732	3.26712	0.00005	0.00781	3.46092	0.0001	0.01351
RASL11A	1.20926	2.86063	6.19499	2.3656	0.0343	0.54697	5.12296	0.0001	0.01351
FMOD	0.85942	3.51664	4.32649	4.09186	0.00065	0.05472	5.03418	0.0001	0.01351

CLSTN3	3.57795	7.78863	12.3256	2.17684	0.0098	0.28701	3.44488	0.0001	0.01351
ANK2	9.19601	22.7836	26.9839	2.47755	0.0022	0.11683	2.93431	0.0001	0.01351
RSPO3	0.62941	4.41782	5.01906	7.01901	0.00015	0.01843	7.97426	0.0001	0.01351
MX1	1.14151	3.55763	5.27772	3.1166	0.0032	0.14835	4.62345	0.0001	0.01351
C1orf115	0.74829	2.85014	3.53166	3.80889	0.00115	0.0759	4.71967	0.0001	0.01351
CCDC68	4.09049	14.8221	18.9988	3.62355	0.00055	0.04936	4.64463	0.0001	0.01351
SEMA3B	5.17733	14.3972	18.4511	2.78082	0.0032	0.14835	3.56383	0.00015	0.01843
COL9A2	0.05686	0.8656	1.48552	15.2232	0.0014	0.0858	26.1256	0.00015	0.01843
MRVI1	8.75456	24.1534	26.5207	2.75895	0.00035	0.03559	3.02936	0.00015	0.01843
WISP1	0.31481	1.75912	1.88472	5.58781	0.0004	0.03952	5.98677	0.00015	0.01843
PTGDS	1.26807	9.82134	12.8061	7.74511	0.00065	0.05472	10.0989	0.00015	0.01843
ID2	36.2159	88.4644	111.648	2.4427	0.0014	0.0858	3.08284	0.00015	0.01843
FAM213A	1.54229	6.31835	11.6113	4.09673	0.00255	0.12857	7.52861	0.00015	0.01843
FGL2	2.13662	17.0177	38.2658	7.96478	0.0036	0.15948	17.9095	0.00015	0.01843
HSD17B7	4.37537	16.3895	20.3716	3.74585	0.0006	0.05203	4.65597	0.00015	0.01843
ZBED3	1.38679	3.24784	4.80499	2.34198	0.0093	0.28025	3.46483	0.00015	0.01843
ADAMTS14	0.99088	2.22644	2.95576	2.24693	0.01015	0.29304	2.98297	0.00015	0.01843
PRRT3	1.21031	4.07025	4.35077	3.36298	0.0007	0.05725	3.59476	0.00015	0.01843
ANTXR1	48.514	98.4778	130.287	2.02988	0.00845	0.26663	2.68555	0.00015	0.01843
OLFML2A	1.19185	4.82345	5.80781	4.04703	0.00105	0.07202	4.87294	0.00015	0.01843
PIR	1.929	7.35318	8.99477	3.81191	0.00125	0.0795	4.66292	0.0002	0.02302
RASSF2	1.86194	5.74684	5.77593	3.08648	0.0003	0.03149	3.1021	0.0002	0.02302
RARRES2	0.40621	4.8783	7.67515	12.0093	0.00085	0.06557	18.8946	0.0002	0.02302
KLHL24	7.03433	14.2111	26.8495	2.02025	0.04065	0.60246	3.81692	0.0002	0.02302
SLC4A3	0.56613	2.40904	3.01715	4.25525	0.00115	0.0759	5.32939	0.0002	0.02302
HRK	0.44937	1.38566	2.40675	3.08356	0.0116	0.3107	5.35583	0.0002	0.02302
CDKL2	0.71619	3.83392	5.39026	5.35322	0.0012	0.07825	7.5263	0.0002	0.02302
CMYA5	0.74393	2.00188	3.132	2.69096	0.00915	0.27761	4.21008	0.0002	0.02302
HSPB3	2.0152	7.00139	11.2631	3.47429	0.00675	0.22958	5.58907	0.0002	0.02302
ARHGEF37	0.59349	2.18305	2.89268	3.67832	0.001	0.06987	4.87401	0.0002	0.02302
NDRG4	1.31144	3.23902	4.52862	2.46982	0.00385	0.16605	3.45317	0.00025	0.02717
PARD3B	3.2124	7.82425	8.93585	2.43564	0.0015	0.0899	2.78167	0.00025	0.02717
CCDC170	0.13169	1.04602	1.7599	7.94287	0.0019	0.10548	13.3637	0.00025	0.02717
PLXDC2	1.43314	4.17422	6.81632	2.91264	0.0103	0.29373	4.75621	0.00025	0.02717
RNF112	0.72123	3.70569	5.26586	5.13804	0.0021	0.11324	7.30125	0.00025	0.02717
IDH1	83.1234	171.744	216.101	2.06613	0.0039	0.16717	2.59976	0.00025	0.02717
VLDLR	3.94341	8.56573	12.7494	2.17216	0.01595	0.36831	3.23309	0.00025	0.02717
ALS2CL	0.374	1.93683	2.77198	5.17863	0.00135	0.08379	7.41163	0.00025	0.02717
LYNX1	3.78173	9.01081	17.0594	2.38272	0.03385	0.54316	4.511	0.00025	0.02717
PROS1	11.073	29.1637	35.6465	2.63377	0.0013	0.08151	3.21923	0.00025	0.02717
RNASE4	3.68167	14.2807	20.6505	3.87886	0.00275	0.13504	5.609	0.00025	0.02717
HSD17B14	3.08701	10.189	13.803	3.3006	0.002	0.10917	4.47132	0.0003	0.03149
HIP1R	3.14901	7.3259	10.3723	2.32641	0.00885	0.27291	3.29383	0.0003	0.03149
CYP27A1	3.65009	7.90203	11.5116	2.16489	0.0156	0.36297	3.15379	0.0003	0.03149
LSS	62.0951	155.793	186.019	2.50894	0.0029	0.1396	2.99571	0.0003	0.03149
ACOX2	2.07837	6.05247	7.31636	2.91212	0.00315	0.14757	3.52024	0.0003	0.03149
C1S	44.1932	99.6325	134.094	2.25448	0.0062	0.21973	3.03427	0.0003	0.03149
WFDC1	0.49661	2.68857	3.5407	5.41389	0.0011	0.07421	7.1298	0.00035	0.03559
GCH1	1.54708	3.3293	6.48808	2.15199	0.04985	0.66414	4.19376	0.00035	0.03559
PLXNC1	0.35719	3.4929	9.5092	9.77877	0.00315	0.14757	26.6221	0.00035	0.03559
GPR37	3.67545	10.344	11.8859	2.81435	0.0014	0.0858	3.23386	0.00035	0.03559
MITF	14.4797	34.29	38.7307	2.36814	0.00345	0.15595	2.67483	0.00035	0.03559
DIO2	0.20061	0.90004	1.84214	4.48648	0.0096	0.28438	9.18265	0.00035	0.03559
PPARGC1A	0.76125	3.14761	5.79519	4.1348	0.01175	0.31266	7.61275	0.0004	0.03952
PLEKHG1	0.10348	0.35196	1.27963	3.40129	0.04645	0.64613	12.3661	0.0004	0.03952
SLC25A23	7.52948	15.6829	20.1676	2.08287	0.0068	0.23047	2.67849	0.0004	0.03952

FRZB	1.66625	4.35599	6.37736	2.61425	0.01175	0.31266	3.82737	0.0004	0.03952
LRRN4CL	6.43874	19.2156	18.3549	2.98437	0.0002	0.02302	2.8507	0.0004	0.03952
PNRC1	9.99771	20.4207	34.5723	2.04254	0.043	0.62073	3.45802	0.00045	0.04367
DGKI	0.96915	4.14991	4.92525	4.28199	0.00145	0.08799	5.08201	0.00045	0.04367
IGSF10	0.16452	1.86226	2.23606	11.3191	0.00445	0.18047	13.5912	0.0005	0.04658
KCNT2	0.403	2.50464	3.17946	6.21499	0.00145	0.08799	7.88948	0.0005	0.04658
JAG1	3.29298	8.44214	9.26271	2.56368	0.00215	0.11505	2.81287	0.00055	0.04936
GJA3	2.39508	6.78254	7.27054	2.83186	0.001	0.06987	3.03561	0.00055	0.04936
CLIC2	0.78525	3.97338	6.19925	5.05999	0.00425	0.17556	7.89458	0.00055	0.04936
STARD5	2.51252	7.82264	9.14957	3.11346	0.00265	0.13185	3.64159	0.00055	0.04936
**Downregulated Genes**									
	**FPKM**			**Nintedanib 2 μM vs. Control**			**Nintedanib 4 μM vs. Control**		
**Gene symbol**	**Control**	**Nintedanib 2 μM**	**Nintedanib 4 μM**	**Ratio**	***P* value**	**FDR *P***	**Ratio**	***P* value**	**FDR *P***
ANLN	258.314	122.378	60.7231	0.47376	0.0035	0.15633	0.23507	0.00005	0.00781
DDX11	12.2006	5.86651	3.00897	0.48084	0.02495	0.46897	0.24662	0.00005	0.00781
TACC3	54.8816	20.6665	11.9089	0.37657	0.0005	0.04658	0.21699	0.00005	0.00781
POLA2	33.6864	13.5076	7.48132	0.40098	0.00405	0.17046	0.22209	0.00005	0.00781
DEPDC1	62.9083	30.1108	17.248	0.47865	0.00835	0.26433	0.27418	0.00005	0.00781
SPDL1	45.9456	22.5936	11.4282	0.49175	0.0181	0.3975	0.24873	0.00005	0.00781
POLQ	16.5807	6.68109	3.25722	0.40294	0.00395	0.1677	0.19645	0.00005	0.00781
NDC1	36.1103	16.4829	10.9186	0.45646	0.00485	0.19068	0.30237	0.00005	0.00781
POLD1	14.0479	6.38862	3.67671	0.45477	0.0062	0.21973	0.26173	0.00005	0.00781
MCM10	10.1315	3.36627	1.49887	0.33226	0.00055	0.04936	0.14794	0.00005	0.00781
ASPM	81.28	36.7945	19.7049	0.45269	0.0019	0.10548	0.24243	0.00005	0.00781
HMMR	62.1595	28.8315	18.3378	0.46383	0.00455	0.1833	0.29501	0.00005	0.00781
MCM2	27.5669	13.4136	6.5804	0.48658	0.0115	0.30989	0.23871	0.00005	0.00781
GTSE1	34.3892	13.3642	7.24551	0.38862	0.0009	0.06867	0.21069	0.00005	0.00781
WDR62	12.8729	4.73551	2.45733	0.36787	0.0013	0.08151	0.19089	0.00005	0.00781
KIF22	40.8674	17.8514	8.91083	0.43681	0.0024	0.12395	0.21804	0.00005	0.00781
NDC80	37.845	16.8787	8.99199	0.446	0.00375	0.1639	0.2376	0.00005	0.00781
ORC1	6.66593	2.69545	1.3049	0.40436	0.00505	0.19494	0.19576	0.00005	0.00781
AURKA	110.059	43.6564	28.2755	0.39666	0.0019	0.10548	0.25691	0.00005	0.00781
TPX2	128.194	51.5882	28.0993	0.40242	0.00035	0.03559	0.21919	0.00005	0.00781
BIRC5	86.4693	37.791	22.1393	0.43705	0.0053	0.19955	0.25604	0.00005	0.00781
ORC6	22.8919	10.0956	5.4266	0.44101	0.0106	0.29675	0.23705	0.00005	0.00781
WDR76	29.6972	14.0233	6.72667	0.47221	0.00715	0.23805	0.22651	0.00005	0.00781
CLSPN	27.1368	11.5398	5.10651	0.42525	0.01925	0.40774	0.18818	0.00005	0.00781
MCM5	49.5594	23.304	11.4927	0.47022	0.00195	0.1074	0.2319	0.00005	0.00781
GINS1	18.9844	8.86539	3.89068	0.46698	0.01165	0.31135	0.20494	0.00005	0.00781
E2F1	8.65258	3.52958	1.49645	0.40792	0.00385	0.16605	0.17295	0.00005	0.00781
FAM83D	28.3336	13.1388	6.84066	0.46372	0.004	0.16894	0.24143	0.00005	0.00781
SUV39H1	10.6833	4.10882	2.45545	0.3846	0.00215	0.11505	0.22984	0.00005	0.00781
ASF1B	14.545	6.442	2.4839	0.4429	0.007	0.23497	0.17077	0.00005	0.00781
NCAPG	71.5707	27.1537	15.2215	0.3794	0.00275	0.13504	0.21268	0.00005	0.00781
RAD51AP1	25.1176	10.4357	4.89066	0.41547	0.0066	0.22748	0.19471	0.00005	0.00781
TIMELESS	21.364	9.47143	5.31377	0.44334	0.00225	0.11897	0.24873	0.00005	0.00781
CDCA3	49.7432	22.4491	9.9378	0.4513	0.021	0.42777	0.19978	0.00005	0.00781
TTK	46.739	18.8182	9.96659	0.40262	0.00105	0.07202	0.21324	0.00005	0.00781
LMNB1	59.8516	21.9989	11.1988	0.36756	0.00005	0.00781	0.18711	0.00005	0.00781
SMC4	283.837	103.333	53.3935	0.36406	0.00025	0.02717	0.18811	0.00005	0.00781
TBCE	5.87129	0.7912	0.71889	0.13476	0.00005	0.00781	0.12244	0.00005	0.00781
CDC20	111.106	42.0052	18.8377	0.37806	0.00025	0.02717	0.16955	0.00005	0.00781
CENPF	86.7	36.18	21.3436	0.4173	0.0003	0.03149	0.24618	0.00005	0.00781
KIF14	21.9062	9.77126	5.45653	0.44605	0.00285	0.13828	0.24909	0.00005	0.00781

TMPO	133.546	57.6289	32.7785	0.43153	0.00115	0.0759	0.24545	0.00005	0.00781
NCAPH	28.8147	10.708	5.54802	0.37162	0.0005	0.04658	0.19254	0.00005	0.00781
PLAU	196.962	69.5404	44.5489	0.35307	0.00005	0.00781	0.22618	0.00005	0.00781
ZWINT	42.9829	17.5512	7.83972	0.40833	0.0011	0.07421	0.18239	0.00005	0.00781
CENPK	72.0429	32.9213	14.9021	0.45697	0.013	0.33029	0.20685	0.00005	0.00781
HJURP	47.5707	22.0644	10.6287	0.46382	0.00325	0.14953	0.22343	0.00005	0.00781
MCM8	23.8815	8.88793	4.85349	0.37217	0.0006	0.05203	0.20323	0.00005	0.00781
DLGAP5	62.6614	28.8159	17.0174	0.45987	0.0038	0.1652	0.27158	0.00005	0.00781
KNSTRN	48.9221	22.2524	13.2892	0.45485	0.00465	0.18595	0.27164	0.00005	0.00781
E2F8	3.86947	1.78133	0.72966	0.46036	0.0296	0.50885	0.18857	0.00005	0.00781
DNMT1	61.3034	29.9001	18.4551	0.48774	0.00235	0.12227	0.30105	0.00005	0.00781
TOP2A	134.235	58.9056	31.1796	0.43882	0.0011	0.07421	0.23228	0.00005	0.00781
CCNB1	185.893	81.109	44.9223	0.43632	0.00085	0.06557	0.24166	0.00005	0.00781
CDCA8	40.2709	14.4036	6.67342	0.35767	0.00005	0.00781	0.16571	0.00005	0.00781
ESPL1	13.1477	4.7175	2.4126	0.35881	0.0074	0.2429	0.1835	0.00005	0.00781
CKAP2	126.951	61.0353	42.5575	0.48078	0.0041	0.17183	0.33523	0.00005	0.00781
NUSAP1	95.2683	41.262	25.9165	0.43311	0.0045	0.18204	0.27204	0.00005	0.00781
KIF23	112.386	52.5193	26.6564	0.46731	0.0126	0.32537	0.23719	0.00005	0.00781
KNL1	52.3554	20.5609	11.1204	0.39272	0.0005	0.04658	0.2124	0.00005	0.00781
KIF11	64.032	28.1985	15.3156	0.44038	0.00185	0.10329	0.23919	0.00005	0.00781
KIF20B	56.7174	21.9501	12.6248	0.38701	0.0005	0.04658	0.22259	0.00005	0.00781
SEMA7A	65.5567	32.2764	21.4335	0.49234	0.0058	0.21233	0.32695	0.00005	0.00781
CENPE	51.5965	20.3309	11.6501	0.39404	0.00055	0.04936	0.22579	0.00005	0.00781
PLK4	37.8082	15.2131	7.88604	0.40238	0.01065	0.29764	0.20858	0.00005	0.00781
DTL	25.5802	10.3995	4.94015	0.40654	0.00255	0.12857	0.19312	0.00005	0.00781
CDCA7	14.3653	6.56852	3.19404	0.45725	0.0129	0.32953	0.22234	0.00005	0.00781
FANCD2	16.5059	6.40219	3.80448	0.38787	0.0006	0.05203	0.23049	0.00005	0.00781
CDCA5	35.2601	13.7044	6.94271	0.38867	0.0008	0.06301	0.1969	0.00005	0.00781
NCAPG2	62.4157	29.8785	16.5515	0.4787	0.00465	0.18595	0.26518	0.00005	0.00781
MKI67	69.6679	28.506	14.9794	0.40917	0.0001	0.01351	0.21501	0.00005	0.00781
HMGA2	55.9092	27.6596	18.4522	0.49472	0.0059	0.21406	0.33004	0.00005	0.00781
CENPU	45.9244	20.9673	12.1179	0.45656	0.01415	0.34864	0.26387	0.00005	0.00781
BUB1B	49.6586	20.7093	11.3828	0.41703	0.0007	0.05725	0.22922	0.00005	0.00781
TONSL	5.13885	2.04179	0.98776	0.39732	0.00215	0.11505	0.19221	0.00005	0.00781
RECQL4	10.3548	3.9389	1.93586	0.38039	0.0038	0.1652	0.18695	0.00005	0.00781
RACGAP1	107.265	53.5641	31.7421	0.49936	0.0051	0.19594	0.29592	0.00005	0.00781
CCNF	11.2753	4.5745	2.06284	0.40571	0.0013	0.08151	0.18295	0.00005	0.00781
PAQR4	13.7954	5.40827	3.06804	0.39203	0.01345	0.33769	0.2224	0.00005	0.00781
OLFML2B	20.4218	8.42408	7.20627	0.4125	0.0005	0.04658	0.35287	0.00005	0.00781
PTX3	70.5403	30.3829	14.7174	0.43072	0.00315	0.14757	0.20864	0.00005	0.00781
CDC25A	9.00041	3.26118	1.90784	0.36234	0.0034	0.1544	0.21197	0.00005	0.00781
ABCA1	15.3357	1.95532	0.95489	0.1275	0.00005	0.00781	0.06227	0.00005	0.00781
SKA3	46.1549	19.4547	10.2887	0.42151	0.00445	0.18047	0.22292	0.00005	0.00781
E2F7	50.7585	17.8079	12.0179	0.35084	0.0001	0.01351	0.23677	0.00005	0.00781
PLK1	83.9217	29.7415	16.0804	0.3544	0.00005	0.00781	0.19161	0.00005	0.00781
CDT1	15.9909	5.08053	1.77095	0.31771	0.0054	0.20222	0.11075	0.00005	0.00781
C19orf48	28.6077	11.9447	7.24645	0.41753	0.00425	0.17556	0.2533	0.00005	0.00781
DTYMK	46.3322	22.7052	13.3662	0.49005	0.01205	0.31788	0.28849	0.00005	0.00781
RFWD3	30.4182	12.5496	9.22672	0.41257	0.0005	0.04658	0.30333	0.00005	0.00781
CKAP2L	35.6476	15.1983	7.82551	0.42635	0.00065	0.05472	0.21952	0.00005	0.00781
BUB1	78.515	33.0343	17.3455	0.42074	0.00095	0.06987	0.22092	0.00005	0.00781
HAS2	375.887	157.688	73.8355	0.41951	0.00095	0.06987	0.19643	0.00005	0.00781
SHCBP1	74.5917	31.0923	17.0354	0.41683	0.0017	0.09793	0.22838	0.00005	0.00781
NPTX1	128.101	52.3086	44.3123	0.40834	0.0005	0.04658	0.34592	0.00005	0.00781
RRM2	228.939	75.0456	33.0058	0.3278	0.00005	0.00781	0.14417	0.00005	0.00781
RGMB	179.477	68.1882	38.9781	0.37993	0.0001	0.01351	0.21718	0.00005	0.00781

EXO1	20.3848	9.75947	4.67617	0.47876	0.0232	0.45116	0.22939	0.00005	0.00781
UBE2C	62.3206	25.9768	16.7051	0.41683	0.00315	0.14757	0.26805	0.00005	0.00781
ATAD5	7.39604	3.12632	1.4731	0.4227	0.0102	0.29345	0.19917	0.00005	0.00781
KCTD12	75.4107	26.2909	24.5638	0.34864	0.00005	0.00781	0.32573	0.00005	0.00781
THBD	15.1317	2.57596	2.13578	0.17024	0.00005	0.00781	0.14115	0.00005	0.00781
AURKB	50.162	20.2259	9.12828	0.40321	0.00245	0.12534	0.18198	0.00005	0.00781
FJX1	17.3493	7.04686	4.06202	0.40618	0.00115	0.0759	0.23413	0.00005	0.00781
KPNA2	243.78	118.083	77.3577	0.48438	0.0044	0.17918	0.31733	0.00005	0.00781
IQGAP3	32.9804	13.8802	6.83349	0.42086	0.0018	0.10189	0.2072	0.00005	0.00781
CDCA2	21.9866	9.99821	4.91137	0.45474	0.0073	0.24075	0.22338	0.00005	0.00781
KIF18B	18.6226	6.78565	2.92914	0.36438	0.0016	0.09372	0.15729	0.00005	0.00781
SAPCD2	5.10629	1.76787	1.03876	0.34621	0.0008	0.06301	0.20343	0.00005	0.00781
H2AFX	108.692	43.9223	20.429	0.4041	0.00055	0.04936	0.18795	0.00005	0.00781
XRCC2	10.1468	3.54788	1.92657	0.34966	0.00155	0.09222	0.18987	0.00005	0.00781
MMP1	1592.81	509.757	406.024	0.32004	0.00005	0.00781	0.25491	0.00005	0.00781
PLXNA4	7.11983	1.32168	1.02647	0.18563	0.00005	0.00781	0.14417	0.00005	0.00781
TMEM158	177.295	71.1591	62.8778	0.40136	0.00045	0.04367	0.35465	0.00005	0.00781
BOP1	16.1359	6.77117	4.66545	0.41963	0.0023	0.12083	0.28913	0.00005	0.00781
UHRF1	32.9605	12.1159	5.59898	0.36759	0.0001	0.01351	0.16987	0.00005	0.00781
CDC7	18.0307	8.79066	4.91336	0.48754	0.0228	0.44765	0.2725	0.0001	0.01351
KPNB1	300.639	149.579	114.378	0.49754	0.00595	0.21508	0.38045	0.0001	0.01351
BRCA2	16.0601	8.01225	4.96521	0.49889	0.00975	0.28588	0.30916	0.0001	0.01351
CCNA2	74.8592	34.8919	22.3802	0.4661	0.01225	0.32073	0.29896	0.0001	0.01351
SGO2	42.3985	16.6445	9.82742	0.39257	0.0045	0.18204	0.23179	0.0001	0.01351
FAM111B	24.5408	10.8848	4.97064	0.44354	0.01185	0.31395	0.20255	0.0001	0.01351
BLM	8.80792	3.50544	2.07012	0.39799	0.00425	0.17556	0.23503	0.0001	0.01351
NCAPD2	45.2882	21.2377	15.5194	0.46895	0.0097	0.28579	0.34268	0.00015	0.01843
TRPA1	108.242	40.5574	31.5867	0.37469	0.00075	0.06003	0.29182	0.00015	0.01843
MCM4	97.245	38.9208	24.3436	0.40023	0.0016	0.09372	0.25033	0.00015	0.01843
NEK2	56.6653	22.1206	13.9535	0.39037	0.0015	0.0899	0.24624	0.00015	0.01843
ODF2	23.7148	10.224	7.93134	0.43112	0.00155	0.09222	0.33445	0.00015	0.01843
FANCA	21.7978	9.09249	3.97189	0.41713	0.01825	0.39918	0.18222	0.00015	0.01843
HNRNPAB	122.388	58.3441	42.5791	0.47671	0.00735	0.24191	0.3479	0.00015	0.01843
PRC1	136.605	60.3391	36.4032	0.4417	0.0086	0.26876	0.26649	0.00015	0.01843
POLE2	14.5055	7.14704	3.05471	0.49271	0.0352	0.55606	0.21059	0.0002	0.02302
NOP56	92.2134	42.7893	29.5816	0.46402	0.00865	0.26963	0.3208	0.0002	0.02302
PARP2	22.0594	10.8126	7.09285	0.49016	0.0227	0.44659	0.32153	0.0002	0.02302
ESM1	29.9752	10.7619	8.11675	0.35903	0.0019	0.10548	0.27078	0.0002	0.02302
LIN9	11.2757	5.25107	2.62941	0.4657	0.0256	0.47552	0.23319	0.0002	0.02302
RFC3	23.1975	11.394	5.96854	0.49117	0.0263	0.47942	0.25729	0.00025	0.02717
POLE3	48.9117	23.2908	15.747	0.47618	0.00805	0.25699	0.32195	0.00025	0.02717
MAD2L1	68.7436	27.6855	16.5247	0.40274	0.0055	0.20471	0.24038	0.0003	0.03149
FANCB	11.5295	3.66456	1.4849	0.31784	0.01185	0.31395	0.12879	0.0003	0.03149
EPHB2	14.4386	6.75201	5.25942	0.46764	0.0077	0.25006	0.36426	0.00035	0.03559
MIS18A	17.9841	8.0902	4.38868	0.44985	0.02095	0.42728	0.24403	0.00035	0.03559
TRIP13	34.4687	14.1606	7.86474	0.41082	0.01835	0.40047	0.22817	0.0004	0.03952
KIF4A	27.111	11.2	6.65858	0.41312	0.0069	0.2329	0.2456	0.00045	0.04367
PHF19	41.1678	17.829	11.8048	0.43308	0.01775	0.39353	0.28675	0.00045	0.04367
TYMS	100.384	45.8894	25.4505	0.45714	0.0245	0.46426	0.25353	0.00045	0.04367
DEPDC1B	10.99	3.33998	2.51667	0.30391	0.00175	0.1001	0.229	0.0005	0.04658
HMGA1	773.782	362.742	304.489	0.46879	0.0048	0.18978	0.39351	0.0005	0.04658
FANCM	7.79188	3.3319	1.98929	0.42761	0.01585	0.36756	0.2553	0.0005	0.04658
SPRY2	38.6608	18.4946	14.9988	0.47838	0.0061	0.21824	0.38796	0.00055	0.04936
NUF2	51.6295	21.4956	10.9253	0.41634	0.01295	0.32953	0.21161	0.00055	0.04936

Table S3. Possible binding sites of the 5 potential miRNA-mRNA interactions.

	Predicted Consequential Pairing of Target Region (Top) and miRNA (Bottom)	Site Type	Context++ Score	Context++ Score Percentile	Weighted Context++ Score
Position 263-269 of ***DDX11*** 3' UTR hsa-miR-486-3p	5' ...GGAGAAGCAGGCAGCCUGCCCCU...<br>||||||| 3' UAGGACAUGACUCGACGGGGC	7mer-m8	−0.09	43	−0.09
Position 1296-1302 of ***DDX11*** 3' UTR hsa-miR-486-3p	5' ...CCUCCAGACAACAUCCUGCCCCU...<br>||||||| 3' UAGGACAUGACUCGACGGGGC	7mer-m8	−0.21	75	−0.21
Position 2823-2829 of ***DDX11*** 3' UTR hsa-miR-486-3p	5' ...UCCCACCUGCAACAGCUGCCCCU...<br>||||||| 3' UAGGACAUGACUCGACGGGGC	7mer-m8	−0.21	76	−0.21
Position 682-689 of ***E2F1*** 3' UTR hsa-miR-486-3p	5' ...CCCUGAGCUGUUCUU--CUGCCCCA...<br>|||| ||||||| 3' UAGGACAUGACUCGACGGGGC	8mer	−0.41	96	−0.38
Position 819-825 of ***E2F1*** 3' UTR hsa-miR-486-3p	5' ...AGUUCAGGGCCCCAGCUGCCCCC...<br>||||||||| 3' UAGGACAUGACUCGACGGGGC	7mer-m8	−0.02	15	−0.02
Position 961-968 of ***E2F1*** 3' UTR hsa-miR-486-3p	5' ...UGCCUCCCCACUGCUCUGCCCCA...<br>|||| ||||||| 3' UAGGACAUGACUCGACGGGGC	8mer	−0.42	96	−0.38
Position 1868-1875 of ***PLXNA4*** 3' UTR hsa-miR-486-3p	5' ...UGUGUGUGUGUGUGUCUGCCCCA...<br>||||||| 3' UAGGACAUGACUCGACGGGGC	8mer	−0.06	37	−0.06
Position 4142-4149 of ***PLXNA4*** 3' UTR hsa-miR-486-3p	5' ...CCCAGCCCUCUGUGACUGCCCCA...<br>||||||| 3' UAGGACAUGACUCGACGGGGC	8mer	−0.03	23	−0.02
Position 4731-4737 of ***PLXNA4*** 3' UTR hsa-miR-486-3p	5' ...CCUGGCUUCUCCCCAUGCCCCAA...<br>|||||| 3' UAGGACAUGACUCGACGGGGC	7mer-A1	−0.06	36	−0.03
Position 5762-5768 of ***PLXNA4*** 3' UTR hsa-miR-486-3p	5' ...GCUUGGCUCUCUGGGCUGCCCCU...<br>||||||| 3' UAGGACAUGACUCGACGGGGC	7mer-m8	−0.02	15	−0.01
Position 65-71 of ***SLC25A23*** 3' UTR hsa-miR-92a-1-5p	5' ...CUGGAGACUGAUGAUCCAACCAC..<br>|||||| 3' UCGUAACGUUGGCUAGGGUUGGA	7mer-A1	−0.15	65	−0.15
Position 136-143 of ***SLC25A23*** 3' UTR hsa-miR-92a-1-5p	5' ...CCUAGACUCCUAUGCCCCAACCA...<br>||||||| 3' UCGUAACGUUGGCUAGGGUUGGA	8mer	−0.33	93	−0.33
Position 194-201 of ***SLC25A23*** 3' UTR hsa-miR-92a-1-5p	5' ...CCUAGACUCCUAUGCCCCAACCA...<br>||||||| 3' UCGUAACGUUGGCUAGGGUUGGA	8mer	−0.29	90	−0.29

Position 480-487 of *SLC25A23* 3' UTR hsa-miR-92a-1-5p	5' ...AAUGGAUCCCAACAC--CCCAACCA...<br>|||| ||||||| 3' UCGUAACGUUGGCUAGGGUUGGA	8mer	−0.35	94	−0.35
Position 580-586 of *SLC25A23* 3' UTR hsa-miR-92a-1-5p	5' ...GCUGGAUCCUAGAUCCCCAACCC...<br>||||||| 3' UCGUAACGUUGGCUAGGGUUGGA	7mer-m8	−0.16	66	−0.16
Position 232-238 of *NPTX1* 3' UTR hsa-miR-486-3p	5' ...GGACUCUCUCAGGCAUGCCCCAU...<br>|||||| 3' UAGGACAUGACUCGACGGGGC	7mer-A1	−0.14	57	−0.14
Position 803-810 of *NPTX1* 3' UTR hsa-miR-486-3p	5' ...CCAGCUCAGCUAGCUCUGCCCCA...<br>||||||| 3' UAGGACAUGACUCGACGGGGC	8mer	−0.21	75	−0.20
Position 864-870 of *NPTX1* 3' UTR hsa-miR-486-3p	5' ...CUGCAAGGCUGGGGUCUGCCCCU...<br>||||||| 3' UAGGACAUGACUCGACGGGGC	7mer-m8	−0.02	15	−0.02
Position 2012-2018 of *NPTX1* 3' UTR hsa-miR-486-3p	5' ...CCGCACCUGCAUGGACUGCCCCG...<br>||||||| 3' UAGGACAUGACUCGACGGGGC	7mer-m8	−0.05	33	−0.05
Position 2393-2399 of *NPTX1* 3' UTR hsa-miR-486-3p	5' ...ACCGCAGACCACCUUCUGCCCCC...<br>||||||| 3' UAGGACAUGACUCGACGGGGC	7mer-m8	−0.05	32	−0.05
Position 2583-2589 of *NPTX1* 3' UTR hsa-miR-486-3p	5' ...ACCUUUAGCUCCCUCCUGCCCCU...<br>||||||| 3' UAGGACAUGACUCGACGGGGC	7mer-m8	−0.15	60	−0.14
